# Incidence of Occult Carcinoma and High-Risk Lesions in Mammaplasty Specimens

**DOI:** 10.1155/2012/145630

**Published:** 2012-10-03

**Authors:** Beth C. Freedman, Sharon M. Rosenbaum Smith, Alison Estabrook, Jasminka Balderacchi, Paul I. Tartter

**Affiliations:** Department of Surgery, Comprehensive Breast Center, St. Luke's Roosevelt Hospital Center, 425 West 59th Street, Suite 7A, New York, NY 10019, USA

## Abstract

*Objectives*. To determine the incidence and type of premalignant or malignant changes in mammaplasty specimens and to determine the incidence of these changes according to age distribution. *Methods*. Retrospective database review of patients who underwent a reduction mammaplasty between 1999 and 2009 was performed from pathology records at a single institution. *Results*. 700 patients were identified. Of the 644 patients who had bilateral reductions, 25 (4%) had significant pathologic findings. The likelihood of finding premalignant changes or cancer increased with advancing patient age (0.8 percent for patients <40 years old and 10 percent for patients >60 years old). Of the 56 patients who underwent unilateral mammaplasty, 12 patients (21%) had significant pathologic findings. The incidence of finding premalignant changes or cancer in this population also increased with advancing patient age (0 for patients <40 years old to 25 percent for patients >60 years old). *Conclusions*. When a unilateral mammaplasty is performed to match a breast reconstructed after cancer surgery, the likelihood of identifying premalignant changes or cancer increases more than fourfold. Therefore, one should consider additional radiologic imaging in the preoperative workup of patients with a history of carcinoma prior to undergoing unilateral mammaplasty.

## 1. Background

There were more than 83,000 reduction mammaplasties performed in the United States in 2010 [1]. Numerous studies have reported that breast cancers and high risk lesions are occasionally found in the breast tissue removed during bilateral reduction mammaplasties. Among patients undergoing unilateral mammaplasty to match a reconstructed breast after breast cancer, the incidence of occult malignancy is higher. We investigated the incidence of pathologic findings in reduction mammaplasty specimens in these two groups and then evaluated the incidence in relation to patient age.

## 2. Methods

A retrospective review was performed using the computerized pathology database at St. Luke's-Roosevelt Hospital Center, New York, NY, USA. The database was searched for “mammaplasty” between 1999 and 2009. Patients included in the study underwent either bilateral reduction mammaplasty or unilateral reduction mammaplasty to match a reconstructed breast after breast cancer surgery. Their operative reports and pathology reports were reviewed. Three random sections were taken from specimens of patients undergoing bilateral reduction and up to 8 random sections were taken from specimens of patients undergoing unilateral reduction. High risk lesions included atypical ductal hyperplasia (ADH), atypical lobular hyperplasia (ALH), and lobular carcinoma in situ (LCIS). Carcinoma included ductal carcinoma in situ (DCIS) and infiltrating ductal carcinoma (IDC). The patients were then stratified according to their age at the time of surgery. The patients over age 40 had mammography within a year of the surgery, as well as clinical breast exams before the procedure. Patients were not included in the study if they had malignancy discovered during their preoperative evaluation.

## 3. Results

Seven hundred patients were included in the analysis. Six hundred forty-four patients (92%) had bilateral reduction mammaplasties for symptomatic macromastia, and 56 patients (8%) had unilateral reductions to match a reconstructed breast after mastectomy or wide excision for malignancy. Twenty-five of the 644 bilateral reduction patients (4%) had significant pathologic findings (Table 1). Of the 21 patients with high risk lesions, 7 patients had ADH, 8 had ALH, and 6 had LCIS. Two patients had invasive ductal carcinoma and two patients had DCIS. One of the patients with invasive ductal carcinoma subsequently underwent a modified radical mastectomy and was found to have metastases in eight lymph nodes. The other patient with IDC had a negative sentinel lymph node biopsy and she received no additional treatment. Neither of the patients with DCIS had additional treatment, as both had widely clear margins. Patients with ADH, ALH, and LCIS did not undergo additional tissue sampling.

When the bilateral mammaplasty patients were stratified by age, the findings of high risk lesions and carcinoma increased with age, from 0.8% in patients under 40 to 10% in patients older than age 60 (*χ* ^2^ = 6.32, *P* = 0.012).

Twelve of the 56 patients (21%) who underwent unilateral mammaplasty had significant pathologic findings (Table 2). Seven patients had ADH, 2 had ALH, and 2 had LCIS. Two patients were diagnosed with DCIS. One of these patients subsequently underwent a completion mastectomy. The specimen contained residual DCIS. Follow-up medical records were not available for the other patient with DCIS. When this group of patients was stratified by age, the incidence of finding high risk lesions or cancer also increased with age, from 0 for patients under age 40 to 25 percent for patients over age 60 (*χ* ^2^ = 0.489, *P* = 0.486).

No occult carcinoma was identified in patients under age 40 in either group. The overall incidence of occult carcinoma in patients over age 40 was 1.6% in patients having a bilateral mammaplasty for macromastia and 4.4% for women over 40 undergoing unilateral reduction to match a reconstructed breast after breast cancer surgery. 

## 4. Discussion

In studies published within the last 10 years containing at least 500 patients undergoing reduction mammaplasties for macromastia, the incidence of occult invasive carcinoma ranges from 0.2 to 0.5% [2–5] (Table 3). Many of these studies inappropriately included LCIS in the same category as DCIS. While DCIS is considered stage 0 breast cancer and requires treatment, LCIS is considered a risk factor for the development of invasive cancer in either breast. Eighty percent of patients with LCIS will never develop invasive breast cancer [6]. These studies are also diluted by the inclusion of patients less than 40 years old. If one excludes patients under age 40, the incidence of malignancy ranges from 1.2 to 1.5%, which is comparable to our results (Table 3). The likelihood of finding malignant changes in these patients increases with increased age.

The likelihood of finding malignancy or high risk lesions in depends not only on the age of the patient, but also on the number of random sections taken from the specimen. Ambaye et al. [7] studied 177 bilateral reduction mammaplasty specimens with the customary three random sections, then took eight additional sections from each specimen. DCIS was discovered in the first three sections of two specimens. Two invasive cancers and one additional DCIS were discovered with the additional random sections. Therefore, the true incidence of carcinoma in the bilateral cosmetic reduction specimens from patients over age forty may actually be double what is reported, indicating that some are missed by the conventional three random sections. Despite the best efforts of surgeons to identify these patients preoperatively with updated mammography and MRI, the incidence of occult cancer in patients with a personal history of breast cancer remains fourfold higher than that in patients with no history of breast cancer. 

The challenge in treating patients in whom an incidental carcinoma is identified is that the specimen is often not oriented, so if any margins are positive, one cannot perform a reexcision. A recent study by Ambaye et al. recommended lumpectomy for patients in whom occult carcinoma is identified [7]. However, they do not address the case when the margins are positive. Mastectomy remains the only option for these patients. When invasive carcinoma is identified in mammaplasty specimens, sentinel lymph node biopsies may be performed with reliable accuracy when the tracer is injected above the surgical scar [8, 9]. The other challenge is radiation therapy. Radiotherapy is the standard adjuvant treatment for patients who have undergone a lumpectomy, with a boost given to the lumpectomy cavity. Due to the lack of specimen orientation and the absence of clips and/or a seroma, the location from which the specimen was excised cannot be ascertained. 

In Sweden, 16-year followup of 30,457 women who had breast reduction surgery for symptomatic macromastia observed only 443 breast cancers [10]. This is 30% fewer than predicted from the age-matched incidence of breast cancer among Swedish women who did not have a breast reduction. Though there may be carcinoma or premalignant changes in the removed mammaplasty specimens, fewer women ultimately developed breast cancer. Despite the fact that cancers were likely missed, this did not result in poorer outcomes for the patients. Rather, it appears that the 30 percent decrease in the incidence of cancer may have been due to removal of occult cancers by the reduction.

Similarly, in our patient population, the identification of a significant pathologic finding in the reduction mammaplasty specimens rarely affected the patient's management or clinical outcome. One patient with invasive cancer subsequently had a mastectomy and chemotherapy, and one patient underwent a sentinel lymph node biopsy, which was negative. Among the unilateral reduction patients, one subsequently had a mastectomy for diffuse DCIS. Therefore, the management was changed for 3 of the 700 patients in the study (0.43%). 

What does this all mean? Clearly, the more one looks for cancer in a mammaplasty specimen, the more one is likely to find cancer. With the conventional three random sections of mammaplasty specimens, it is likely that one or two cancers are missed per 100 patients. However, the Swedish study shows that the consequence of missing and not treating these cancers is more than offset by an overall reduction in breast cancer risk among patients undergoing breast reductions.

## 5. Conclusion

Overall, malignancy was noted in 6 of 700 patients (0.86%), and high risk lesions were identified in 31 of the patients (4.4%). Since the likelihood of malignancy is the highest in mammaplasty specimens from patients over age 40 with contralateral malignancy, these patients should undergo more rigorous screening of the breast. This can include a combination of all three available modalities: mammography, ultrasound, and MRI. This will potentially decrease, but not eliminate, missed cancers in mammaplasty specimens. 

## Figures and Tables

**Table 1:** Bilateral reduction mammaplasty.

Age	*N*	Atypical ductal hyperplasia	Atypical lobular hyperplasia	LCIS	DCIS	Infiltrating ductal carcinoma
<40	361	2 (0.55%)	0	1 (0.28%)	0	0
40–49	152	2 (1.31%)	3 (1.97%)	3 (1.97%)	1 (0.66%)	1 (0.66%)
50–59	101	3 (2.97%)	3 (2.97%)	1 (0.99%)	1 (0.99%)	1 (0.99%)
>60	30	0	2 (6.67%)	1 (3.33%)	0	0

**Table 2:** Unilateral mammaplasty after contralateral carcinoma.

Age	*N*	Atypical ductal hyperplasia	Atypical lobular hyperplasia	LCIS	DCIS	Infiltrating ductal carcinoma
<40	11	0	0	0	0	0
40–49	13	2 (15.4%)	0	1 (7.69%)	0	0
50–59	20	3 (15.0%)	0	1 (5.00%)	2 (10.0%)	0
>60	12	1 (8.33%)	2 (16.7%)	0	0	0

**Table 3:** Findings of cancer in bilateral reduction specimens.

Reference	No. of patients	DCIS (%)	Invasive carcinoma (%)	No. of patients over 40 (% with DCIS or invasive carcinoma)
Dotto et al., 2008 [2]	516	1 (0.2)	1 (0.2)	164 (1.2)
Pitanguy et al., 2005 [3]	2488	3 (0.1)	7 (0.3)	602 (1.5)
Colwell et al., 2004 [4]	611	2 (0.3)	2 (0.3)	Not stated
Ishag et al., 2003 [5]	518	1 (0.2)	2 (0.4)	197 (1.5)
